# Elevated Expression of Serum Amyloid A 3 Protects Colon Epithelium Against Acute Injury Through TLR2-Dependent Induction of Neutrophil IL-22 Expression in a Mouse Model of Colitis

**DOI:** 10.3389/fimmu.2018.01503

**Published:** 2018-06-29

**Authors:** Gufang Zhang, Jin Liu, Lehao Wu, Yu Fan, Lei Sun, Feng Qian, Daijie Chen, Richard D. Ye

**Affiliations:** ^1^School of Pharmacy, Shanghai Jiao Tong University, Shanghai, China; ^2^Institute of Chinese Medical Sciences, State Key Laboratory of Quality Research in Chinese Medicine, University of Macau, Macau, China

**Keywords:** serum amyloid A, inflammation, innate immunity, cytokines, colitis, neutrophils

## Abstract

Induced expression of serum amyloid A (SAA) is a hallmark of many inflammatory diseases, but whether SAA exacerbates inflammation or protects tissues against injury remains unclear. In dextran sulfate sodium (DSS)-induced colitis, SAA3 is the predominant isoform of inducible SAA proteins that also include SAA1 and SAA2, and mice with genetic deletion of *Saa3* exhibits increased production of proinflammatory cytokines, decreased expression of IL-22 along with aggravated epithelium disruption, and reduced colon length compared with wild-type littermates. Colonic neutrophils have been identified as a major source of IL-22 in these mice. Administration of exogenous SAA3 as recombinant protein to *Saa3*^−/−^ mice improves neutrophil IL-22 production, colonic epithelial integrity, and secretion of the antimicrobial peptides Reg3β and Reg3γ. Stimulation of mouse bone marrow neutrophils with mouse SAA3 or human SAA1 leads to expansion of IL-22-producing neutrophils. Unlike previously reported IL-22 induction through IL-23, the SAA3-induced neutrophil IL-22 expression utilizes a TLR2-dependent mechanism that does not depend on IL-23. Adoptive transfer of the SAA3-treated neutrophils to *Saa3*^−/−^ mice ameliorates DSS-induced colitis and improves colonic epithelial integrity. These findings suggest that in the DSS-induced mouse colitis model, SAA isoforms are expressed to different extent in colon and deletion of *Saa3* renders these mice more susceptible to DSS-induced injury. The presence of SAA3 in the inflamed colon mucosal serves to protect epithelial barrier in part through expansion of IL-22-producing neutrophils. It is speculated that SAA3 stimulation of autologous neutrophils may have therapeutic potential for inflammatory bowel disease.

## Introduction

The acute-phase response is an evolutionarily conserved biological phenomenon in vertebrates with poorly defined physiological functions (1). Characterized with elevated production of acute-phase proteins including serum amyloid A (SAA) and C-reactive protein, the acute-phase response is triggered by environmental cues such as trauma, microbial infection, malignancy, and severe stress (2). These cues often lack the specificity for adaptive immunity, and some of them also lack features of microbial patterns that induce innate immune response. As a result, our understanding of the cellular mechanisms of the acute-phase response is far from complete.

Up to 1,000-fold induction of SAA expression has been observed in many inflammatory diseases based on DNA array analysis as well as protein level measurement of acute-phase serum and tissue samples (1). The marked induction of SAA expression suggests its possible involvement in the regulation of inflammatory response. SAA is the collected name of four different genes in humans and mice. Of these genes, *Saa1, Saa2*, and *Saa3* encode inducible or acute-phase SAA proteins whereas *Saa4* codes for a constitutively expressed protein. The mechanism for the induced expression of *Saa* at the transcription level has already been delineated, indicating that SAA production results from stimulation by inflammatory cytokines such as IL-1β and IL-6 (2). However, it was unclear until recently whether SAA actively participates in the inflammatory process or simply serves as an inflammatory biomarker. Emerging evidence has shown that SAA possesses cytokine-like activities and induces a multitude of inflammatory cytokines and chemokines including IL-1β, TNFα, and IL-8 (3–5). Moreover, SAA promotes neutrophilia through G-CSF production, serving as an endogenous ligand for neutrophil expansion in asceptic inflammation (6). These findings suggest that SAA contributes to inflammation and innate immunity.

More recent studies have added new evidence for immunoregulatory functions of SAA. Littman and coworkers reported that SAA1/2 are mediators of intestinal T_H_17 response induced by gut microbiome through the IL-23/IL-22 circuit (7). SAA is known as an endogenous ligand for the induction of IL-23 but not IL-12 (8). In addition to stimulating proinflammatory cytokine expression, SAA serves as a potent inducer of IL-10 expression in phagocytes (9, 10) while promoting M2 macrophage differentiation (11). These seemingly paradoxical observations reflect the diverse functions of SAA [reviewed in Ref. (12)], suggesting that the *in vivo* functions of SAA may be more than promotion of inflammation.

We hypothesize that inducible expression of SAA at the organ-environment interface, such as in the respiratory system and gastrointestinal tract, may contribute to host defense and local immunity. Indeed, studies have shown the presence of SAA at inflammatory sites in human intestine (13, 14) and in mouse colitis models (7, 15). An important yet unanswered question is whether inducible expression of SAA exacerbates local inflammation or protects intestinal epithelium from further attack by environmental factors. Built upon previous findings of SAA expression in the intestinal epithelium, the present study uses dextran sulfate sodium (DSS)-induced mouse colitis as a model for investigation of the potential involvement of acute-phase SAA in the homeostasis of colonic epithelial barrier. Our study led to the findings that inducible expression of SAA3 by colonic epithelial cells instigates changes in lamina propria (LP) and promotes neutrophil IL-22 secretion that protects mice against intestinal epithelial injury. These findings provide a new mechanism by which an acute-phase protein serves to enhance host defense and local immunity.

## Materials and Methods

### Laboratory Animals

*Saa3*^−/−^ mice were generated by Knockout Mice Project Repository (Davis, CA, USA), and maintained on C57BL/6 genetic background. Animals were kept at Shanghai Jiao Tong University Laboratory Animal Center under specific pathogen-free conditions until 7–10 weeks. Age- and sex-matched knockout and wild type (WT) littermates were used in all experiments. All animal experiments were conducted at Shanghai Jiao Tong University with procedures approved by the Biological Research Ethics Committee of Shanghai Jiao Tong University, China.

### DSS-Induced Colitis and Colitis Evaluation

Experimental colitis in mice was induced with 3.5% (w/v) DSS salt (MP Biomedicals, Solon, OH, USA), provided *ad libitum* in drinking water. *Saa3*^−/−^ and WT littermates were sacrificed at indicated days after DSS treatment for measurements of weight change, colon length, and colon histology. Colon length from the end of the cecum to the anus was measured. For gene expression studies, colons were removed, immediately frozen in liquid nitrogen, and stored at −80°C until use. For survival assays, mice were given DSS for 7 days followed with normal drinking water until day 15.

For histology analysis, colons were removed, fixed in 4% polyoxymethylene overnight, paraffin-embedded, and sectioned. Tissue sections were stained with hematoxylin and eosin (H&E) for microscopic examination. The severity of tissue injury and inflammation were analyzed in a blinded manner. Epithelia barrier injury score was 0 = normal morphology, 1 = loss of goblet cell, 2 = loss of goblet cells in large areas, 3 = loss of crypts, and 4 = loss of crypts in large areas. Infiltration of leukocytes score was 0 = no infiltrate, 1 = infiltrate around crypt bases, 2 = infiltrate reaching to muscularis mucosae, 3 = extensive infiltration reaching the muscularis mucosae and thickening of the mucosae with abundant edema, and 4 = infiltration to the submucosa.

For PAS staining, the paraffin-embedded tissue sections (5 µm) were deparaffinized and hydrated, then placed in Schiff reagent for 15 min followed by counterstaining in hematoxylin for 1 min. The excess dye was washed with water and the section was dehydrated on neutral resin coverslips.

### Immunofluorescence and Immunochemistry

Frozen colon sections (7 µm) were incubated with an anti-pan-keratin (1:200) overnight at 4°C and then an Alex Fluor^®^488 donkey anti-mouse antibody (1:500; Invitrogen) for 1 h at room temperature. The sections were then incubated with an anti-SAA3 (1:500) overnight at 4°C followed by incubation with an Alex Fluor^®^555 donkey anti-rabbit IgG (1:500; Invitrogen) for 1 h at room temperature. Sections were counterstained with DAPI for 10 min. Fluorescent images were captured using a laser-scanning confocal fluorescence microscope (TCS SP8, Leica Microsystems).

### Isolation of LP Cells

Mice were sacrificed and colons were excised immediately. Colons were opened longitudinally and the content was removed. Colon tissues were cut into 5 mm in length and incubated with 5 mM EDTA plus 1 mM DTT at 37°C in a shaker with 150 rpm for 15 min and the tissues were then minced. LP cells were isolated by incubation for 40 min at 37°C with RPMI 1640 containing 5% FBS, 1 mg/ml collagenase VIII, and 20 µg/ml dispase (Sigma-Aldrich). The cells were collected by passing through a 40-µm diameter filter and centrifuged at 350 × *g* for 10 min. The pellet was resuspended and LP cells were collected from the interface of a 40 and 80% Percoll gradient by centrifugation.

### Flow Cytometry

Colonic LP cells and bone marrow neutrophils were surface stained with antibodies for 30 min at 4°C before flow cytometry analysis. Intracellular protein staining was carried out with cells cultured in RPIM 1640 containing GolgiPlug™ for the last 4 h before harvest. The cells were then fixed and permeabilized as instructed (eBioscience, San Diego, CA, USA). The cells were resuspended in PBS for flow cytometry analysis (BD LSR FORTESSA, BD Biosciences, New York, NY, USA).

### Neutrophil Isolation and Adoptive Transfer

Total bone marrow cells from mice were flushed from femurs and tibias with PBS (Ca^2+^, Mg^2+^ free) and cell suspension was passed through a 40-µm diameter filter. After removal of erythrocytes by ACK lysis buffer, the remaining cells were resuspended in PBS and put onto 72% Percoll and Nyco-prep for density gradient equilibrium centrifugation. Neutrophils were collected from the interface of 72% Percoll and Nyco-prep. Neutrophils from WT mice were cultured with recombinant SAA3 (rSAA3) in UltraCulture medium (GIBICO). After 6 h, the cells were harvested and 5 × 10^5^ neutrophils were adoptively transferred *via* tail vein into *Saa3*^−/−^ mice 1 day after DSS treatment. Colitis was assessed as described above.

### Quantitative RT-PCR

Total RNA was isolated from colon tissue using an RNA extraction kit (Takara, Beijing, China) and from neutrophils using Trizol reagent (Invitrogen, CA, USA). cDNA was generated using PrimeScript™ RT Master Mix (Takara) and used as template for quantitative RT-PCR using SYBR Green Master Mix (TOYOBO, Shanghai, China) on an Applied Biosystems 7500 Fast Real-Time PCR System. The gene expression level was calculated relative to GAPDH expression level.

### Detection of SAA3 Protein

Mouse colon tissue was homogenized in lysis buffer containing 20 mM HEPES, 420 mM NaCl, 1.5 mM MgCl_2_, 1 mM DTT, 1 mM PMSF, 0.5 mM EDTA, and phosphatase inhibitor cocktails (Sigma-Aldrich). Protein concentrations were determined with BCA protein Assay (YESEN Biotechnology, Shanghai, China). An appropriate volume of 5× SDS-PAGE sample buffer was added and samples were denatured by heating to 99°C for 10 min. Samples were separated on 15% SDS-PAGE and transferred onto nitrocellulose membranes. The blots were incubated with primary antibodies to mouse SAA3 and β-actin (1:1,000) overnight at 4°C followed by an anti-rabbit IRDye 800CW secondary antibody. The blots were detected by Odyssey CLX Infrared Imaging System, the density of protein bands were measured using the NIH Image J software. The intensity of the SAA3 species was normalized against that of β-actin that was used as an internal control.

### Intestinal Tissue Culture

Distal colon tissue (0.5 cm in length) was cut into pieces and cultured in 0.5 ml RPMI 1640 containing 10% FBS, penicillin–streptomycin, and gentamycin (100 µg/ml). The tissue was macroscopically examined during culture to ensure proper culture condition. The supernatant was collected after 24 h and stored at −80°C for IL-22 quantification by ELISA (eBioscience, MA, USA).

### Expression and Purification of Recombinant Mouse SAA3

The cDNA encoding mouse SAA3 (without the signal peptide sequence) was amplified by PCR and cloned into pET28a (+) plasmid between *NdeI* and *BamHI* restriction endonuclease sites, with an N-terminal hexahistidine tag and a C-terminal stop codon added. Protein expression in the *E. coli* strain BL21 (DE3) was conducted as described previously (16). To minimize endotoxin contamination, all glass containers are sterilized by dry heat at 250°C for 30 min, and injection-grade water is used throughout final purification steps for buffer preparation. After a repeating step of Ni^2+^ affinity chromatography and a subsequent desalting, the resulting protein product was frozen at −80°C in aliquots until use. The endotoxin level was below 0.05 ng/µg of SAA3 protein as determined using by a Tachypleus amebocyte lysate kit (Zhanjiang A&C Biological, Guangdong, China).

### Statistical Analysis

Unpaired Student’s *t*-test was used for two-group comparison and analysis of variance was used for sample analysis with more than two groups. The Kaplan–Meier methods and log-rank test was used to analyze cumulative survival rate. Data are presented as mean ± standard margin of error. All experiments were performed for at least three times with sample size indicated in the figure legends. A *p* value less than 0.05 was considered statistically significant. Data were plotted using the Prism software (GraphPad, San Diego, CA, USA).

## Results

### SAA3 Expression Is Highly Inducible in DSS Colitis

**Figure 1:**
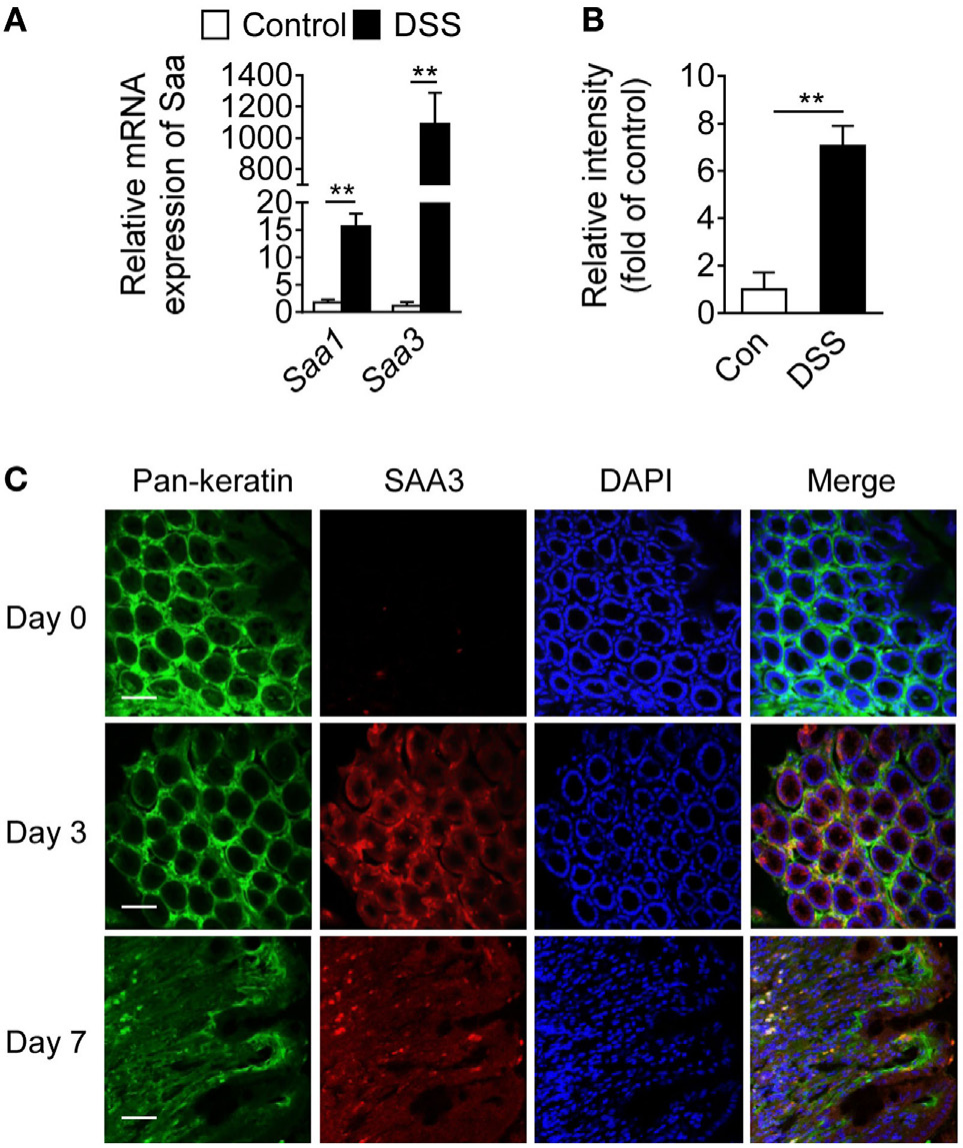
Serum amyloid A (SAA)3 expression in dextran sulfate sodium (DSS)-induced mouse colitis model. **(A)** Relative mRNA expression levels of *Saa1* and *Saa3* in colonic tissue of wild-type (WT) mice treated with control (water only) or DSS (3.5% in water), based on quantitative RT-PCR [mean ± standard margin of error (SEM), *n* = 5, triplicate]. **(B)** Relative expression levels of SAA3 protein in colonic tissue of WT mice without (open bar) and with (closed bar) 3.5% DSS treatment, based on Western blot analysis (mean ± SEM, *n* = 5, triplicate). **(C)** Representative immunofluorescence images showing SAA3 protein expression (red) in mouse colonic epithelium (green) before (day 0) and 3 or 7 days after giving 3.5% DSS in drinking water (*n* = 5). Scale bar, 20 µm. **p < 0.01 between groups. Male and female mice 8–10 weeks of age were used in all experiments.

Serum amyloid A 3 is often found in inflammatory tissues in mice, but its role in inflammatory diseases remains incompletely understood. Following DSS stimulation, colon mucosal SAA3 expression level was ~60-fold higher than that of SAA1 at the mRNA level on day 7 (Figure 1A). SAA3 protein level was eightfold above baseline in the colonic mucosa after DSS treatment (Figure 1B). Immunofluorescent staining of colonic tissue (Figure 1C) found that the induced SAA3 proteins (red fluorescence) co-localized with epithelial cells (pan-keratin positive, green fluorescence). These results indicate that SAA3 is the predominant isoform of inducible SAA proteins in DSS-treated colonic epithelium. To further evaluate the *in vivo* functions of SAA3 in the pathogenesis of DSS-induced colitis, *Saa3*^−/−^ mice were generated as described in Section “Materials and Methods” and illustrated in Figure S1A in Supplementary Material. Immunoblotting analysis of colonic tissue extracts identified no SAA3 expression at the protein level when the *Saa3*^−/−^ mice were challenged with DSS (Figure S1B in Supplementary Material).

### Genetic Deletion of Saa3 Exacerbates DSS-Induced Colitis

**Figure 2:**
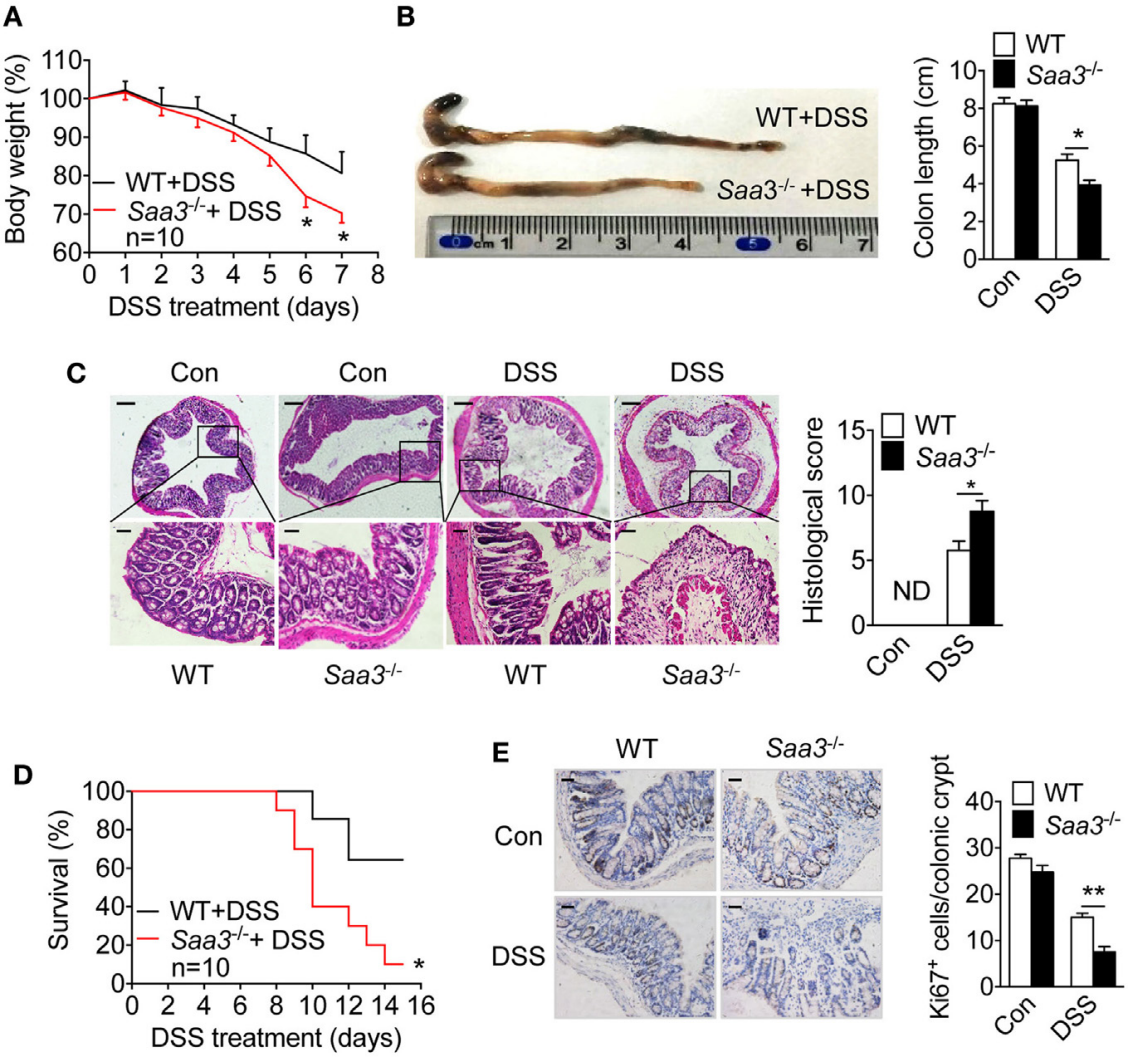
Dextran sulfate sodium (DSS)-induced pathological changes in wild type (WT) and *Saa3*^−/−^ mice. Male and female mice 8–10 weeks of age were used in the experiments. **(A)** Body weight change in WT (black) and *Saa3*^−/−^ (red) mice (*n* = 10) receiving 3.5% DSS in drinking water for up to 7 days. **(B)** Mice were sacrificed on day 7 and colons were excised. Macroscopic examination of colon lengths in WT and *Saa3*^−/−^ mice (left) and statistical analysis of colon lengths (right, *n* = 5). **(C)** Representative images of hematoxylin and eosin stained colon sections from control and DSS-treated mice after 7 days. Scale bars, 50 and 20 micrometers in upper and lower panels, respectively. The histological scores of colon pathology are shown in the right panel (*n* = 5). **(D)** Survival rates of mice treated with 3.5% DSS for 7 days followed by normal drinking water for another 8 days (*n* = 10 each). **(E)** Representative images showing proliferative cells (Ki67^+^) in colon of mice with DSS-induced colitis. Quantification of Ki67^+^ cells in each crypt is shown in right panel (*n* = 5). Scale bars, 20 micrometers. All quantitative data are mean ± standard margin of error based on triplicate measurements. *p < 0.05 and **p < 0.01 between WT and *Saa3*^−/−^ mice.

The pathological changes in the *Saa3*^−/−^ mice were compared with those in WT littermates after DSS treatment. Progressive weight loss was observed in both strains of mice, but starting from day 5 weight loss began to accelerate in the *Saa3*^−/−^ mice (Figure 2A). Seven days after DSS treatment, the *Saa3*^−/−^ mice showed significantly shortened colon length compared with their WT littermates (Figure 2B). Histological analysis of H&E stained colonic tissue identified more severe damage to epithelium in the *Saa3*^−/−^ mice than in their WT littermates, with complete loss of crypts 7 days after DSS administration (Figure 2C). In survival assays, death occurred to the *Saa3*^−/−^ mice on day 8, which was 2 days earlier than their WT littermates (Figure 2D). By day 15, approximately 90% of the *Saa3*^−/−^ mice were dead, compared to 40% of the WT littermates. In addition to disrupting colonic epithelium, DSS treatment changed the dynamics of colonic epithelial cell regeneration. The number of crypt progenitor cells, stained positive with the proliferation marker Ki67, was significantly lower in the *Saa3*^−/−^ mice than in their WT littermates (Figure 2E).

**Figure 3:**
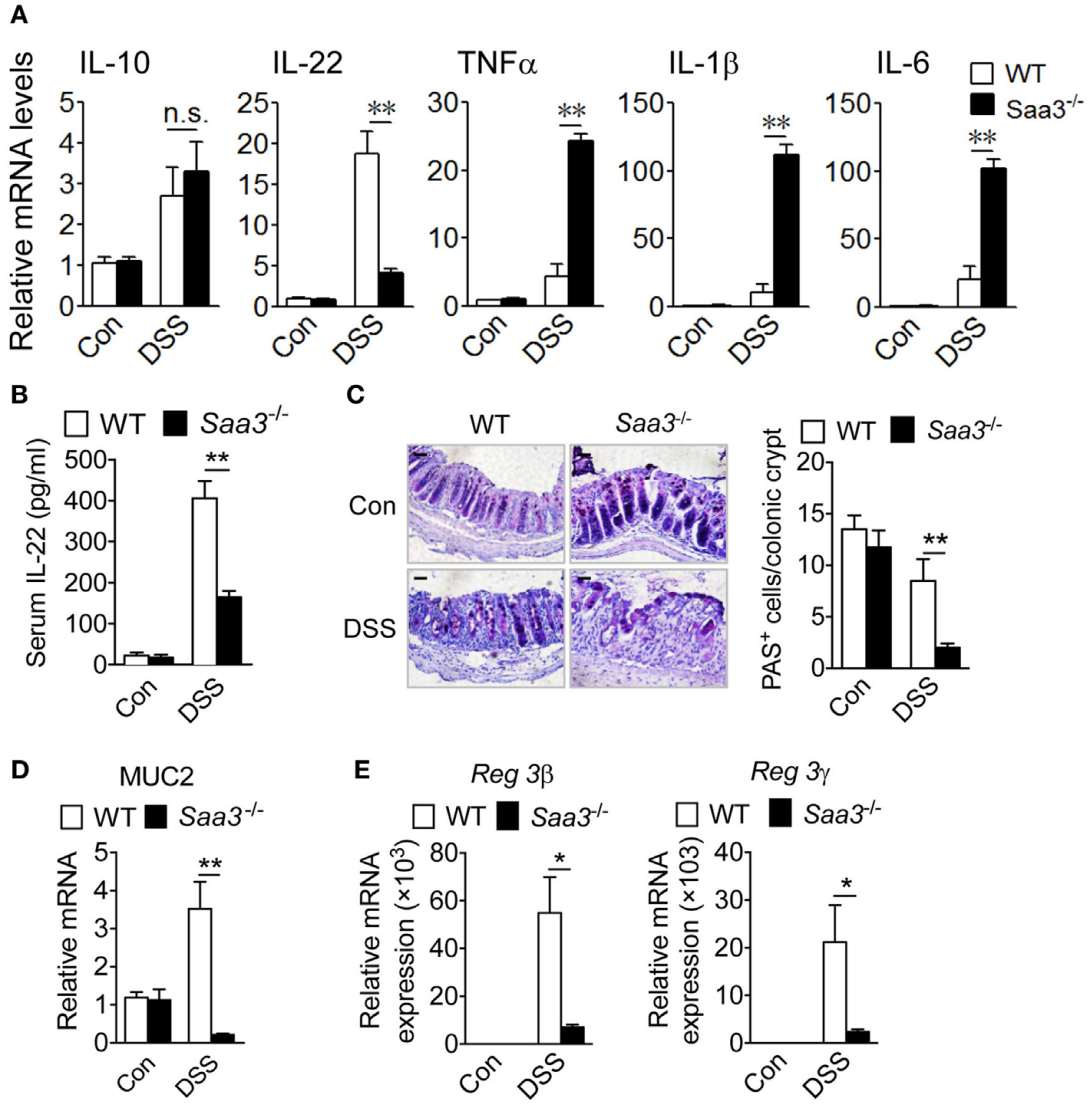
Expression of cytokines, mucin, and antimicrobial peptides in wild type (WT) and *Saa3*^−/−^ mice receiving dextran sulfate sodium (DSS). **(A)** The relative mRNA levels of regulatory cytokines (IL-10 and IL-22) and inflammatory cytokines (TNFα, IL-1β, and IL-6) were measured with quantitative RT-PCR. **(B)** Changes in serum IL-22 levels WT and *Saa3*^−/−^ mice. **(C)** Representative images showing PAS^+^ goblet cells in colon sections from WT and *Saa3*^−/−^ mice treated with DSS or water. Scale bars, 20 µm. Quantitative data (*n* = 5) are shown on the right. **(D)** Relative mRNA levels of MUC2 in WT and *Saa3*^−/−^ mice. **(E)** Relative mRNA levels of Reg3β (left) and Reg3γ (right) in the colon tissue from WT and *Saa3*^−/−^ mice. All data above were obtained using male and female mice (*n* = 5) of 8–10 weeks of age that were treated for 7 days with DSS or water. Shown in bar charts above are mean ± standard margin of error of triplicate measurements. *p < 0.05 and **p < 0.01; n.s., non-significant.

Cytokines produced in the LP constitute a network that influence the functions of neighboring cells. Loss of *Saa3* produced no significant changes in colonic expression of IL-10 upon DSS treatment, but local production of IL-22 was significantly reduced in *Saa3*^−/−^ mice (Figure 3A). Likewise, serum IL-22 level in *Saa3*^−/−^ mice were significantly lower than that in WT mice (Figure 3B). IL-22 is a member of the IL-10 cytokine family, and many published studies report that it helps to maintain the integrity of colon epithelium (17, 18). Along with the reduced IL-22 expression, there was a significant induction in the proinflammatory cytokines TNF-α, IL-1β, and IL-6 in *Saa3*^−/−^ colonic tissue (Figure 3A). These findings indicate that the loss of *Saa3* skews the local cytokine production toward a proinflammatory profile.

The integrity of colonic epithelial barrier is maintained in part through mucus production by goblet cells and antimicrobial protein secretion from epithelial cells. In the *Saa3*^−/−^ mice, there was a significant reduction in the number of periodic acid-Schiff (PAS)-positive goblet cells per crypt (Figure 3C). With the loss of goblet cells, there was a significant reduction in MUC2 gene expression in the *Saa3*^−/−^ mice compared with the WT mice (Figure 3D). Moreover, the production of the antimicrobial peptides Reg3β and Reg3γ, known to bind bacteria peptidoglycans and protect host against Gram-positive bacterial infection, was significantly reduced in the absence of *Saa3* (Figure 3E).

**Figure 4:**
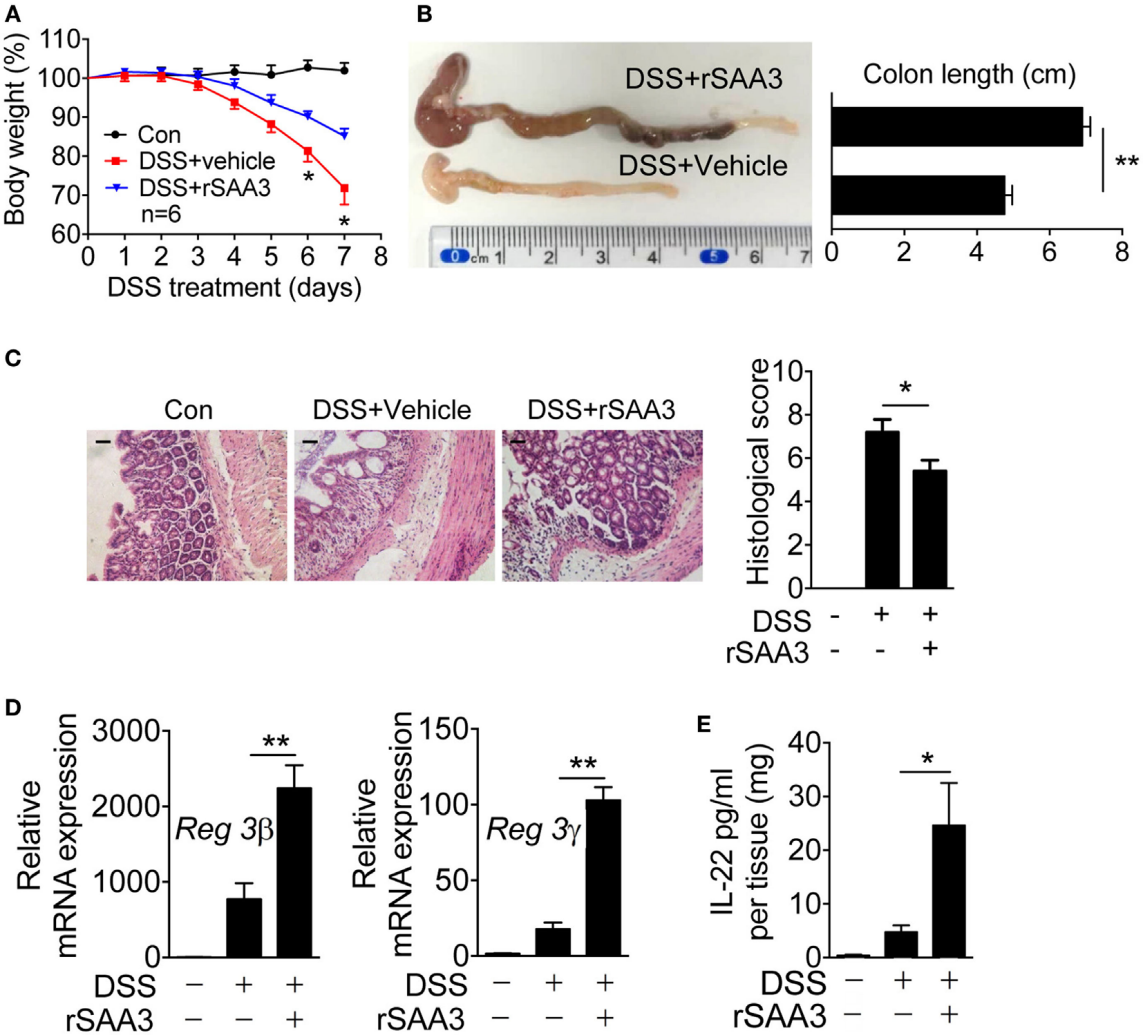
Amelioration of dextran sulfate sodium (DSS)-induced colitis by recombinant SAA3 (rSAA3). **(A)** Weight loss was monitored every day in *Saa3*^−/−^ mice receiving DSS with rSAA3 (blue) or without rSAA3 (red) or drinking water only (black). **(B)** A representative image (left) and quantitative analysis of colon length measurement (right, *n* = 5). **(C)** Representative images of paraffin sections of distal colon tissue were stained with hematoxylin and eosin (left panel), and pathology scores were obtained (right panel, *n* = 5). Scale bars, 20 micrometers. **(D)** Relative mRNA levels of Reg3β (left) and Reg3γ (right) in *Saa3*^−/−^ colitis mice receiving water or DSS with or without rSAA3 were quantitatively analyzed (*n* = 5). **(E)** Colonic tissue explants (*n* = 5 each, receiving water, DSS, and DSS + rSAA3) were collected 7 days after treatment and then cultured *ex vivo* for 24 h for measurement of secreted IL-22 by ELISA. Male and female mice 8–10 weeks of age were used in the above experiments. For all quantitative data above, *p < 0.05 and **p < 0.01 between different groups.

To further confirm that SAA3 is critical to the integrity of colon epithelium, rSAA3 was prepared as described in Section “Materials and Methods” and administered to the *Saa3*^−/−^ mice through intraperitoneal injection (Figure S2 in Supplementary Material). Mice were sacrificed on day 7 and pathological changes in the *Saa3*^−/−^ mice receiving SAA3 or vehicle (PBS) were compared. Administration of rSAA3 to *Saa3*^−/−^ mice significantly reduced the rate of body weight loss (Figure 4A), improved the length of colon compared with the control mice (Figure 4B), and partially reversed DSS-induced damage to the colon epithelium (Figure 4C). Of interest, administration of rSAA3 partially restored Reg3β and Reg3γ expression (Figure 4D) and potentiated IL-22 expression in the colonic tissue of *Saa3*^−/−^ mice receiving DSS (Figure 4E).

### SAA3 Increases the Number of IL-22-Positive Neutrophils

**Figure 5:**
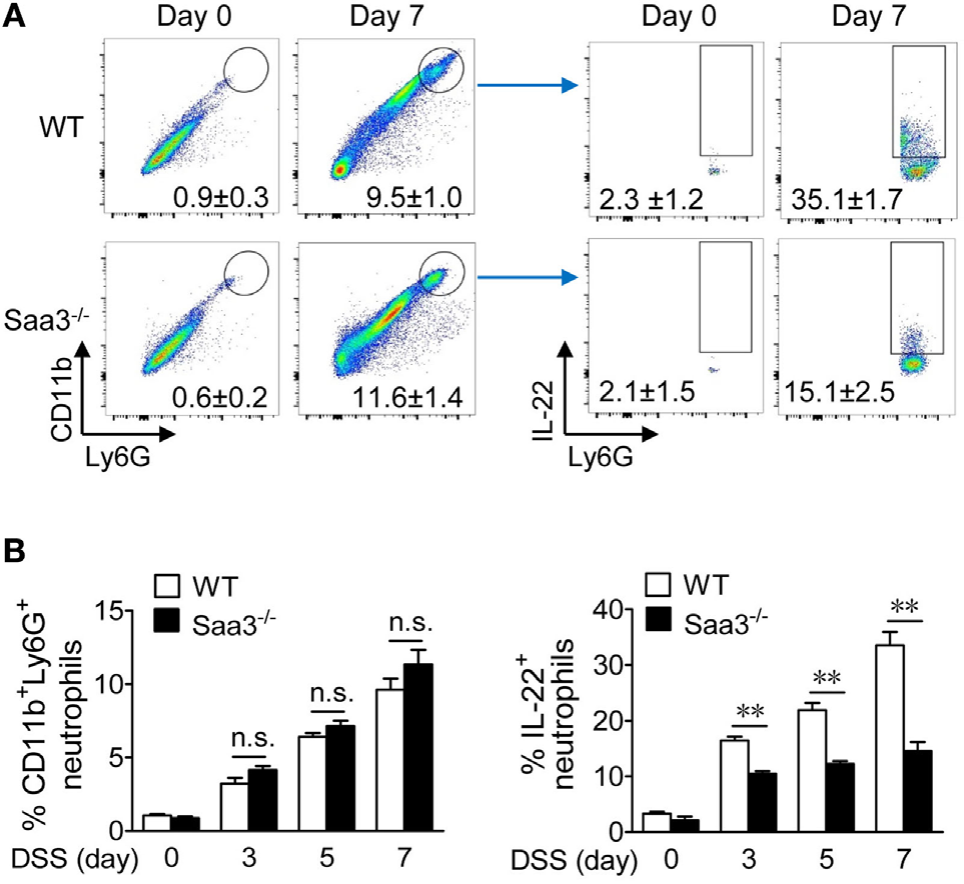
Flow cytometry analysis of colonic neutrophils in wild type (WT) and *Saa3*^−/−^ mice. Male and female mice 8–10 weeks of age were used in the experiments. **(A)** Representative flow cytometry dot plots of colonic neutrophils from WT and *Saa3*^−/−^ mice were determined (left panels). This population of neutrophils was further analyzed by IL-22 staining (right), based on data from five mice in each group treated with dextran sulfate sodium (DSS) for 0, 3, 5, and 7 days. Data collected on day 7 were shown. The quantification results are shown in **(B)** for the percentage of CD11b^+^Ly6G^high^ cells (left) and the percentage of IL-22^+^ neutrophils in that population of neutrophils (right). Shown are mean ± standard margin of error based on three measurements. **p < 0.01 between samples.

In colonic tissues, IL-22 production has been identified in mononuclear leukocytes including macrophages, dendritic cells, innate lymphoid cells (19), and more recently, neutrophils (20). Neutrophil infiltration is an early sign of acute inflammation with both beneficial and deleterious effects (21). To evaluate neutrophil IL-22 production in the presence or absence of SAA3, granulocytes were prepared from LP of WT and *Saa3*^−/−^ mice before (day 0) and after DSS treatment (days 3, 5, and 7) and analyzed by flow cytometry (Figure 5A, day 7 data shown in panels to the left). DSS treatment significantly increased the percentage of neutrophils (CD11b^+^Ly6G^high^) in both WT and *Saa3*^−/−^ mice. This change was seen as early as on day 3 after DSS treatment (Figure 5B). The percentage of IL-22^+^ neutrophils within this cell population also increased markedly in WT mice following DSS treatment for 3, 5, and 7 days (Figure 5A, panels to the right; Figure 5B, panel to the right). In *Saa3*^−/−^ mice, the percentage of IL-22^+^ neutrophils in the CD11b^+^Ly6G^high^ cell population was significantly lower than in WT littermates (*p* < 0.01, Figure 5B).

**Figure 6:**
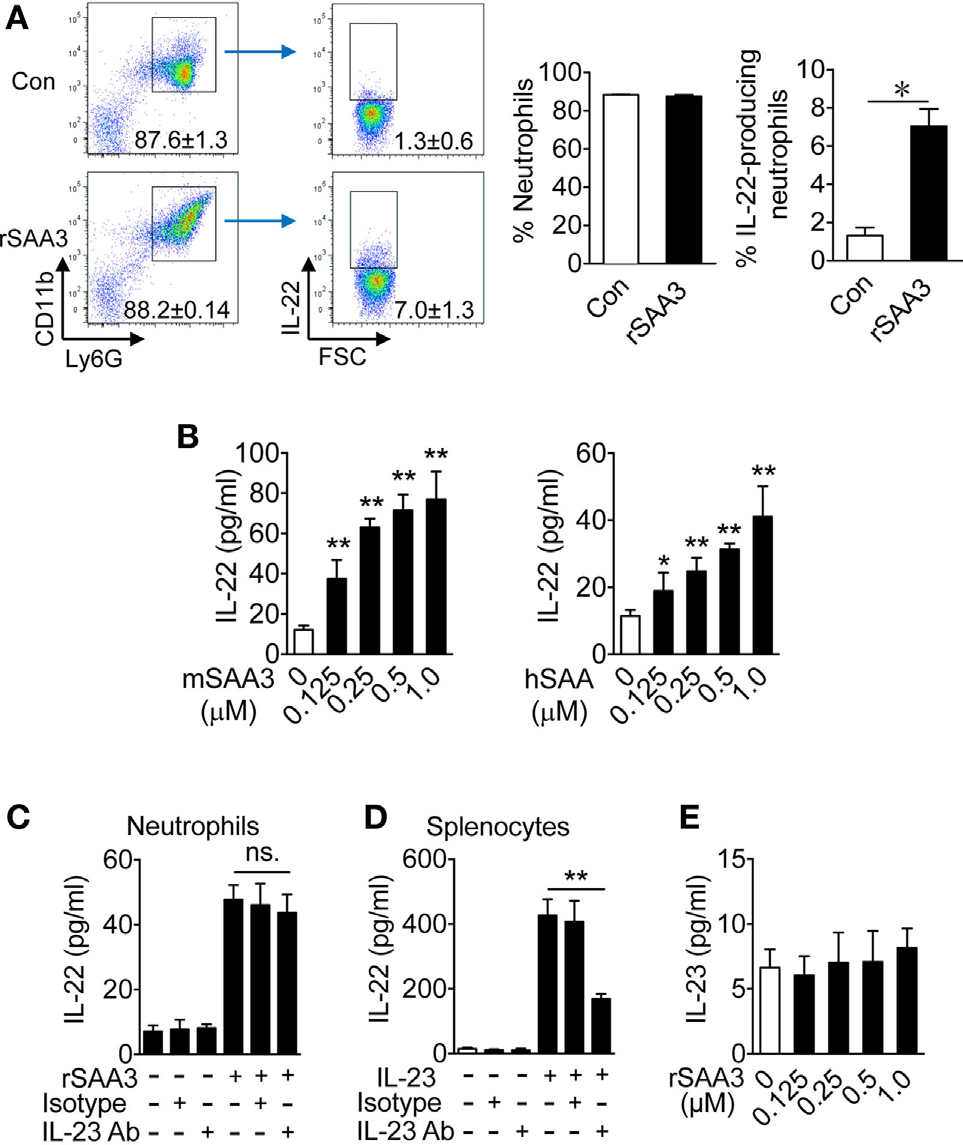
Serum amyloid A (SAA)3 induces neutrophil expression of IL-22. Neutrophils from bone marrow of wild-type C57BL/6 mice (both male and female, 8–10 weeks of age) were treated with recombinant SAA3 (rSAA3) (1 µM) or PBS (Con) for 24 h before analysis. **(A)** Representative flow cytometry dot plots showing the effects of rSAA3 on the CD11b^+^Ly6G^high^ neutrophil population (left two panels) and IL-22-producing neutrophils (right two panels). Quantification data, based on five mice in each group, are shown in bar charts on the right. *p < 0.05. **(B)** Bone marrow neutrophils were treated with mouse rSAA3 (mSAA3, left) or human rSAA1 (hSAA, right) for 24 h. IL-22 release was quantified by ELISA. Bone marrow neutrophils **(C)** or splenocytes **(D)** were incubated with an anti-IL-23 antibody (1 µg/ml) or IgG isotype control (Isotype) for 1 h before adding rSAA3 (1 µM) for 24 h. The culture medium was collected and IL-22 concentration was determined by ELISA. Splenocytes (right) from the same mice were pre-incubated with the IL-23 Ab or IgG isotype control for 1 h before stimulation with 20 ng/ml of IL-23 for 24 h. IL-22 release was measured by ELISA. **(E)** IL-23 production by neutrophils stimulated with indicated concentrations of rSAA3 for 24 h. All quantitative data shown are mean ± standard margin of error based on triplicate measurement, using neutrophils from three to five mice. *p < 0.05, **p < 0.01 between different samples compared.

Given that *Saa3* deletion reduced neutrophil IL-22 production and that neutrophils are major infiltrating leukocytes after DSS-induced epithelial damage, the possibility that SAA3 directly stimulates neutrophil IL-22 production was examined. Bone marrow neutrophils were prepared from WT mice and their IL-22 expression level was determined before and after SAA3 stimulation. In unstimulated cells, very few IL-22^+^ neutrophils were identified in the CD11b^+^Ly6G^high^ cell population (Figure 6A, upper right quadrant). The percentage of IL-22^+^ neutrophils increased significantly upon SAA3 stimulation (1 µM, 24 h; Figure 6A, lower right quadrant and bar chart on the right). These findings suggest positive regulation of neutrophil IL-22 expression by SAA3.

### SAA3-Treated Neutrophils Express Increased Levels of IL-22 but Not IL-23

The ability of recombinant human SAA1 to induce IL-22 expression was compared with that of SAA3. Mouse bone marrow neutrophils were stimulated with either mSAA3 or hSAA1 at the indicated concentrations before measurement of IL-22 production. Both SAA isoforms induced IL-22 secretion in a dose-dependent manner (Figure 6B), suggesting that the ability to induce neutrophil IL-22 production, which is beneficial to the maintenance of intestinal epithelial barrier, is conserved between human SAA1 and mouse SAA3.

We previously reported that SAA is an endogenous ligand for IL-23 production in mononuclear cells (8), raising the possibility that SAA3 and SAA1 might stimulate IL-22 production through the IL-23/IL-22 route, as seen in IL-22 secretion by T_H_17 cells (22) and ILC3 cells (7). To determine whether IL-23 is a major driving force for IL-22 expression in SAA3-stimulated neutrophils, bone marrow neutrophils were pre-incubated with an anti-IL-23 antibody or IgG isotype control for 1 h before SAA3 stimulation. As shown in Figures 6C,D, the anti-IL-23 antibody did not significantly alter SAA3-induced IL-22 production in neutrophils but inhibited IL-22 production by mouse splenocytes that were used as a control for effective inhibition by the anti-IL-23. Moreover, SAA3 did not induce a significant amount of IL-23 production in mouse neutrophils (Figure 6E). These results suggest that the IL-23/IL-22 circuit might be intact in neutrophils but did not contribute as significantly to SAA3-induced IL-22 production in neutrophils as in T_H_17 and ILC3 cells.

### SAA3-Induced Neutrophil IL-22 Expression Requires TLR2

**Figure 7:**
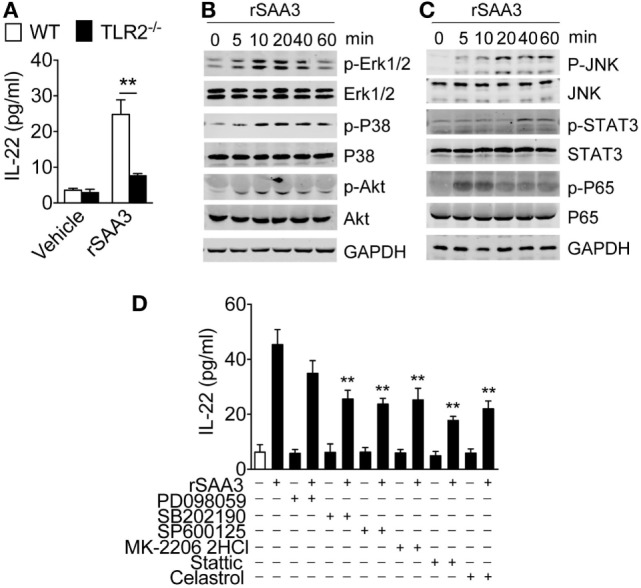
Regulation of serum amyloid A (SAA)3-induced IL-22 expression in neutrophils. **(A)** Bone marrow neutrophils from WT or *Tlr2*^−/−^ mice (both male and female, 8–10 weeks of age) were stimulated with recombinant SAA3 (rSAA3) (1 µM) or vehicle for 24 h. IL-22 release was measured by ELISA. **(B,C)** SAA3-induced phosphorylation of selected signaling molecules. Bone marrow neutrophils were treated with rSAA3 (1 µM) as indicated. Cells were collected at indicated time points and cell lysates were separated on SDS-PAGE for Western blotting using antibodies to the phosphorylated and total proteins of selected signaling molecules (*n* = 3–5 mice for each group). **(D)** Pharmacological inhibition of SAA3-induced IL-22 secretion. Bone marrow neutrophils were pre-treated with the ERK1/2 inhibitor PD98059 (100 µM), p38 inhibitor SB202190 (100 µM), JNK inhibitor SP600125 (10 µM), Akt inhibitor MK-2206 2HCl (10 µM), STAT3 inhibitor static (10 µM), and NF-κB inhibitor celastrol (100 µM) for 1 h before rSAA3 (or medium only) stimulation for 24 h. The released IL-22 was quantified by ELISA (*n* = 3–5 mice for each group).

IL-22 is a member of the IL-10 family of cytokines, and our previous studies showed that IL-10 induction by recombinant SAA was TLR2-dependent (9). Published work has also shown that mouse SAA3 is a ligand of TLR2 and can induce STAT3 phosphorylation through TLR2 (23). To determine whether SAA3-induced IL-22 expression requires TLR2, bone marrow neutrophils were prepared from WT and *Tlr2*^−/−^ mice and stimulated with SAA3. As shown in Figure 7A, deletion of *Tlr2* significantly compromised SAA3-induced IL-22 production. The SAA3-triggered TLR2-dependent signaling pathways leading to IL-22 expression was next examined. SAA3 stimulation of bone marrow neutrophils led to rapid phosphorylation of ERK and p38 MAPK, the serine/threonine kinase Akt, and the NF-κB component p65/RelA (Figure 7B). A slower kinetics was observed with the phosphorylation of JNK and Stat3 that peaked 20–40 min after stimulation (Figure 7C). Treatment of bone marrow neutrophils with pharmacological inhibitors for p38 MAPK (SB202190), JNK (SP600125), Akt (MK-2206 2HCl), Stat3 (stattic), and NF-κB (celastrol) produced partial but significant inhibition of SAA3-induced IL-22 production (Figure 7D). These results indicate that TLR2 signaling plays an important role in neutrophil IL-22 production following SAA3 stimulation.

### SAA3-Stimulated Neutrophils Protect Colonic Epithelium Against DSS-Induced Injury

**Figure 8:**
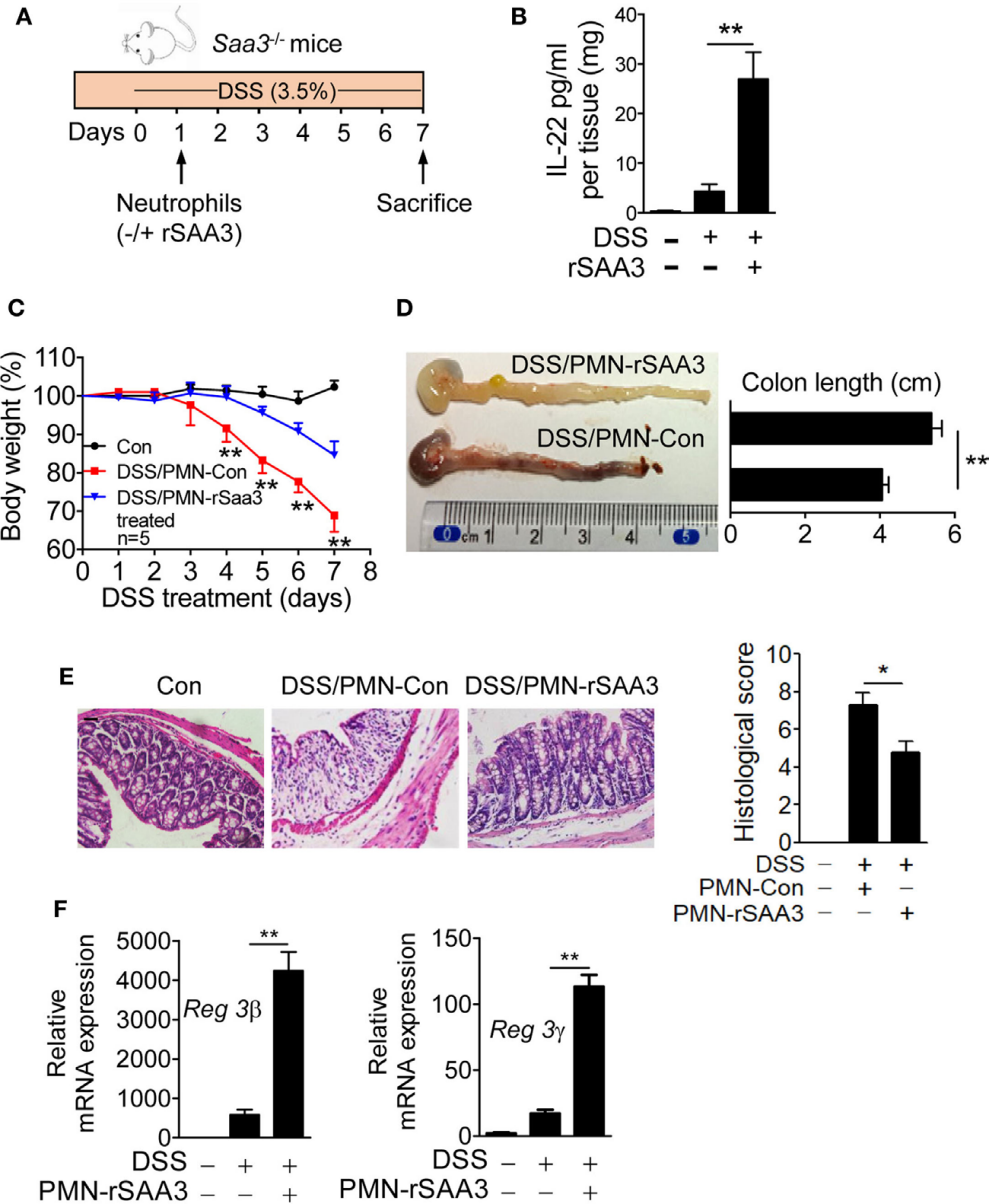
Adoptive transfer of serum amyloid A (SAA)3-treated neutrophils ameliorates dextran sulfate sodium (DSS)-induced colitis. **(A)** Bone marrow neutrophils from wild-type (WT) mice were incubated for 6 h with or without 1 µM of recombinant SAA3 (rSAA3) and 5 × 10^5^ cells were intravenously injected into a Saa3^−/−^ mouse 1 day after DSS administration. **(B)** IL-22 released into the culture medium was measured by ELISA. **(C)** Body weight loss was monitored every day in Saa3^−/−^ mice receiving rSAA3-treated neutrophils (blue) or vehicle-treated neutrophils (red). Untreated controls were included. **(D)** The lengths of colons were measured with a representative image shown on the left (*n* = 5 each condition). **(E)** Representative images of hematoxylin and eosin stained paraffin sections of distal colon tissue (left panels) and pathology scores (right; *n* = 5). Scale bars, 20 µm. **(F)** Relative mRNA levels of the antimicrobial peptides Reg3β (left) and Reg3γ (right) in colon tissue samples. Male and female mice 8–10 weeks of age were used in these experiments. All data were collected with triplicate measurements and shown as mean ± standard margin of error. *p < 0.05 and **p < 0.01.

Bone marrow neutrophils were isolated from WT mice and incubated for 6 h with 1 µM of SAA3 or with medium only. The cells (5 × 10^5^/mouse) were then transferred intravenously to the *Saa3*^−/−^ mice 1 day after DSS administration (Figure 8A). By day 7 the IL-22 expression level in the colon tissue of mice receiving SAA3-treated neutrophils was significantly higher than that in control mice receiving vehicle-treated neutrophils (Figure 8B). Adoptive transfer of SAA3-treated neutrophils but not untreated neutrophils reduced the rate of body weight loss (Figure 8C), partially restored colon length (Figure 8D), and alleviated damage to the crypts (Figure 8E) in *Saa3*^−/−^ mice receiving DSS. Finally, adoptive transfer of SAA3-treated neutrophils improved colonic expression of the antimicrobial peptides Reg3β and Reg3γ (Figure 8F). These results suggest a possible mechanism by which induced expression of SAA3 protects colonic epithelium through neutrophils.

## Discussion

The present study identifies SAA3 as the predominant acute-phase SAA induced in the DSS colitis model, which is characterized with chemical injury to the colonic epithelium and subsequent bacterial infection. The severity of DSS colitis correlates positively with the loss of *Saa3*, and administration of rSAA3 ameliorates epithelial injury. Our results show that colonic epithelium produces large amounts of SAA3 upon DSS stimulation, supporting a hypothesized function of SAA3 in host defense at the organ-environment interface. All three acute-phase SAA isoforms share high degrees of sequence homology and function similarly in several *in vitro* assays, but the difference in their modes of induction may explain why genetic deletion of *Saa3* produces a prominent phenotype. In mice, SAA1 and SAA2 are expressed in normal intestinal epithelium that are exposed to selected gut microbiota including segmented filamentous bacteria (7, 24). In comparison, the expression of SAA3 is low in unstimulated colonic tissue. It is therefore predicted that the constant presence of SAA1/2 in unchallenged mice helps to maintain homeostasis at the intestinal epithelium while the robust induction of SAA3 expression by exposure to environmental insults such as DSS serves mainly to protect intestinal epithelium against acute injury.

Neutrophils are the primary cells found at sites of acute infection and inflammation and are widely known as terminally differentiated leukocytes with specialized bactericidal activities. We observed that adoptive transfer of SAA3-treated neutrophils ameliorates DSS-induced colonic epithelial injury, suggesting that local production of SAA3 by colonic epithelial cells may influence the functions of infiltrating neutrophils. These neutrophils exhibit an altered phenotype characterized with increased IL-22 secretion, enhanced bactericidal activities, and reduced apoptosis. IL-22 was initially identified as an IL-10 family cytokine produced by leukocytes such as macrophages, dendritic cells, and innate lymphoid cells (19). This cytokine has been known for strengthening the barrier functions of intestinal epithelium and induction of antimicrobial peptides in human studies as well as mouse models of colitis (17, 18, 25, 26). Neutrophil production of IL-22 contributes to host defense at the colonic epithelium (20), suggests that these inflammatory cells play a role in tissue homeostasis. Our study extends the findings of the above study through the identification of epithelium-derived SAA3 as a ligand for neutrophil production of IL-22. These neutrophils help to fortify intestinal epithelial barrier against DSS-induced injury.

Combined with genetic deletion of *Saa3* and administration of rSAA3, our *in vivo* studies demonstrate a novel mechanism by which induced expression of SAA3 at intestinal epithelium protects against acute injury in part through expansion of IL-22-positive neutrophils. The ability of SAA3 to induce neutrophil IL-22 production is shared by human SAA1, whose expression in human intestinal epithelium is elevated after exposure to LPS and selected inflammatory cytokines (14). The present study, therefore, suggest a general mechanism for host defense by acute-phase proteins. Unlike what was reported previously on the IL-23/IL-22 circuit (7, 22), we found that induced IL-22 production in SAA3-stimulated neutrophils relies heavily on TLR2 signaling. Although the IL-23/IL-22 circuit may be functional in neutrophils, SAA3 stimulation did not induce a significant increase in IL-23 secretion. These findings suggest that in addition to the SAA/IL-23/IL-22 pathway (8), SAA3 may induce IL-22 expression through TLR2 signaling (9). As shown in the present study, SAA3 was able to induce the phosphorylation of key signaling molecules that are required for IL-22 expression, suggesting that SAA3 activation of TLR2 may be an alternative to the well-established IL-23/IL-22 circuit for IL-22 production.

In summary, the present study reports a mechanism by which the acute-phase protein SAA3 serves to protect against chemical insult to the intestinal epithelium. The findings of this study connect previous reports on IL-22 secretion and protection of intestinal epithelium by identifying SAA3 as a potent endogenous factor upstream of these events. Our findings challenge the dogma that acute-phase SAA is a proinflammatory cytokine that exacerbates colitis, and that infiltrating neutrophils cause damage to colonic tissue. Moreover, the difference in the induced expression of mouse SAA3 vs. SAA1 and SAA2 suggests that this inducible isoform of SAA is more suited for the protection of colon epithelium than maintaining a steady state function. Further studies of SAA3 as a modulator of local immune response will likely advance our understanding of the acute-phase response as a part of the innate immune mechanism. Finally, the present study illustrates the prospect of developing cell-based therapies for inflammatory bowel disease through transfusion of acute-phase SAA-treated autologous neutrophils.

## Ethics Statement

This study was carried out in compliance with the Statute on the Administration of Laboratory Animals by the Ministry of Science and Technology of China. All experiments involving laboratory animals were carried out at Shanghai Jiao Tong University using procedures approved by the Biological Research Ethics Committee of Shanghai Jiao Tong University, China.

## Author Contributions

GZ and RY designed the study; GZ, JL, LW, and YF performed the experiments; GZ and RY analyzed the data and wrote the manuscript; LS, FQ, and DC provided critical guidance and discussions.

## Conflict of Interest Statement

The authors declare that the research was conducted in the absence of any commercial or financial relationships that could be construed as a potential conflict of interest.

## References

[1] Gabay C Kushner I Acute-phase proteins and other systemic responses to inflammation. N Engl J Med (1999) 340(6):448–54. 10.1056/NEJM199902113400607 9971870

[2] Uhlar CM Whitehead AS. Serum amyloid A, the major vertebrate acute-phase reactant. Eur J Biochem (1999) 265(2):501–23. 10.1046/j.1432-1327.1999.00657.x 10504381

[3] Patel H Fellowes R Coade S Woo P. Human serum amyloid A has cytokine-like properties. Scand J Immunol (1998) 48(4):410–8. 10.1046/j.1365-3083.1998.00394.x 9790312

[4] Furlaneto CJ Campa A. A novel function of serum amyloid A: a potent stimulus for the release of tumor necrosis factor-alpha, interleukin-1beta, and interleukin-8 by human blood neutrophil. Biochem Biophys Res Commun (2000) 268(2):405–8. 10.1006/bbrc.2000.2143 10679217

[5] He R Sang H Ye RD. Serum amyloid A induces IL-8 secretion through a G protein-coupled receptor, FPRL1/LXA4R. Blood (2003) 101(4):1572–81. 10.1182/blood-2002-05-1431 12393391

[6] He RL Zhou J Hanson CZ Chen J Cheng N Ye RD. Serum amyloid A induces G-CSF expression and neutrophilia via toll-like receptor 2. Blood (2009) 113(2):429–37. 10.1182/blood-2008-03-139923 18952897 PMC2615655

[7] Sano T Huang W Hall JA Yang Y Chen A Gavzy SJ An IL-23R/IL-22 circuit regulates epithelial serum amyloid A to promote local effector Th17 responses. Cell (2015) 163(2):381–93. 10.1016/j.cell.2015.08.061 26411290 PMC4621768

[8] He R Shepard LW Chen J Pan ZK Ye RD. Serum amyloid A is an endogenous ligand that differentially induces IL-12 and IL-23. J Immunol (2006) 177(6):4072–9. 10.4049/jimmunol.177.6.4072 16951371

[9] Cheng N He R Tian J Ye PP Ye RD. Cutting edge: TLR2 is a functional receptor for acute-phase serum amyloid A. J Immunol (2008) 181(1):22–6. 10.4049/jimmunol.181.1.22 18566366 PMC2464454

[10] De Santo C Arscott R Booth S Karydis I Jones M Asher R Invariant NKT cells modulate the suppressive activity of IL-10-secreting neutrophils differentiated with serum amyloid A. Nat Immunol (2010) 11(11):1039–46. 10.1038/ni.1942 20890286 PMC3001335

[11] Sun L Zhou H Zhu Z Yan Q Wang L Liang Q Ex vivo and in vitro effect of serum amyloid A in the induction of macrophage m2 markers and efferocytosis of apoptotic neutrophils. J Immunol (2015) 194(10):4891–900. 10.4049/jimmunol.1402164 25870242 PMC4417396

[12] Ye RD Sun L. Emerging functions of serum amyloid A in inflammation. J Leukoc Biol (2015) 98(6):923–9. 10.1189/jlb.3VMR0315-080R 26130702 PMC6608020

[13] Meek RL Benditt EP. Amyloid A gene family expression in different mouse tissues. J Exp Med (1986) 164(6):2006–17. 10.1084/jem.164.6.2006 3783088 PMC2188489

[14] Vreugdenhil AC Dentener MA Snoek AM Greve JW Buurman WA. Lipopolysaccharide binding protein and serum amyloid A secretion by human intestinal epithelial cells during the acute phase response. J Immunol (1999) 163(5):2792–8. 10453023

[15] Eckhardt ER Witta J Zhong J Arsenescu R Arsenescu V Wang Y Intestinal epithelial serum amyloid A modulates bacterial growth in vitro and pro-inflammatory responses in mouse experimental colitis. BMC Gastroenterol (2010) 10:133. 10.1186/1471-230X-10-133 21067563 PMC2992040

[16] Chen M Zhou H Cheng N Qian F Ye RD. Serum amyloid A1 isoforms display different efficacy at Toll-like receptor 2 and formyl peptide receptor 2. Immunobiology (2014) 219(12):916–23. 10.1016/j.imbio.2014.08.002 25154907 PMC4252704

[17] Sugimoto K Ogawa A Mizoguchi E Shimomura Y Andoh A Bhan AK IL-22 ameliorates intestinal inflammation in a mouse model of ulcerative colitis. J Clin Invest (2008) 118(2):534–44. 10.1172/JCI33194 18172556 PMC2157567

[18] Sonnenberg GF Fouser LA Artis D. Border patrol: regulation of immunity, inflammation and tissue homeostasis at barrier surfaces by IL-22. Nat Immunol (2011) 12(5):383–90. 10.1038/ni.2025 21502992

[19] Zenewicz LA Flavell RA. Recent advances in IL-22 biology. Int Immunol (2011) 23(3):159–63. 10.1093/intimm/dxr001 21393631

[20] Zindl CL Lai JF Lee YK Maynard CL Harbour SN Ouyang W IL-22-producing neutrophils contribute to antimicrobial defense and restitution of colonic epithelial integrity during colitis. Proc Natl Acad Sci U S A (2013) 110(31):12768–73. 10.1073/pnas.1300318110 23781104 PMC3732935

[21] Fournier BM Parkos CA. The role of neutrophils during intestinal inflammation. Mucosal Immunol (2012) 5(4):354–66. 10.1038/mi.2012.24 22491176

[22] Zheng Y Danilenko DM Valdez P Kasman I Eastham-Anderson J Wu J Interleukin-22, a T(H)17 cytokine, mediates IL-23-induced dermal inflammation and acanthosis. Nature (2007) 445(7128):648–51. 10.1038/nature05505 17187052

[23] Lee JM Kim EK Seo H Jeon I Chae MJ Park YJ Serum amyloid A3 exacerbates cancer by enhancing the suppressive capacity of myeloid-derived suppressor cells via TLR2-dependent STAT3 activation. Eur J Immunol (2014) 44(6):1672–84. 10.1002/eji.201343867 24659444

[24] Atarashi K Tanoue T Ando M Kamada N Nagano Y Narushima S Th17 cell induction by adhesion of microbes to intestinal epithelial cells. Cell (2015) 163(2):367–80. 10.1016/j.cell.2015.08.058 26411289 PMC4765954

[25] Zenewicz LA Yancopoulos GD Valenzuela DM Murphy AJ Stevens S Flavell RA. Innate and adaptive interleukin-22 protects mice from inflammatory bowel disease. Immunity (2008) 29(6):947–57. 10.1016/j.immuni.2008.11.003 19100701 PMC3269819

[26] Zheng Y Valdez PA Danilenko DM Hu Y Sa SM Gong Q Interleukin-22 mediates early host defense against attaching and effacing bacterial pathogens. Nat Med (2008) 14(3):282–9. 10.1038/nm1720 18264109

