# Supplementary material for: Elevated Expression of Serum Amyloid A 3 Protects Colon Epithelium Against Acute Injury Through TLR2-Dependent Induction of Neutrophil IL-22 Expression in a Mouse Model of Colitis
Source: Front Immunol. 2018 Jun 29;9:1503. doi: 10.3389/fimmu.2018.01503 (PMC6033967; doi:10.3389/fimmu.2018.01503)
Supplement: Supplementary file 1 [file Image_1.PDF]

Supplemental Figures S1

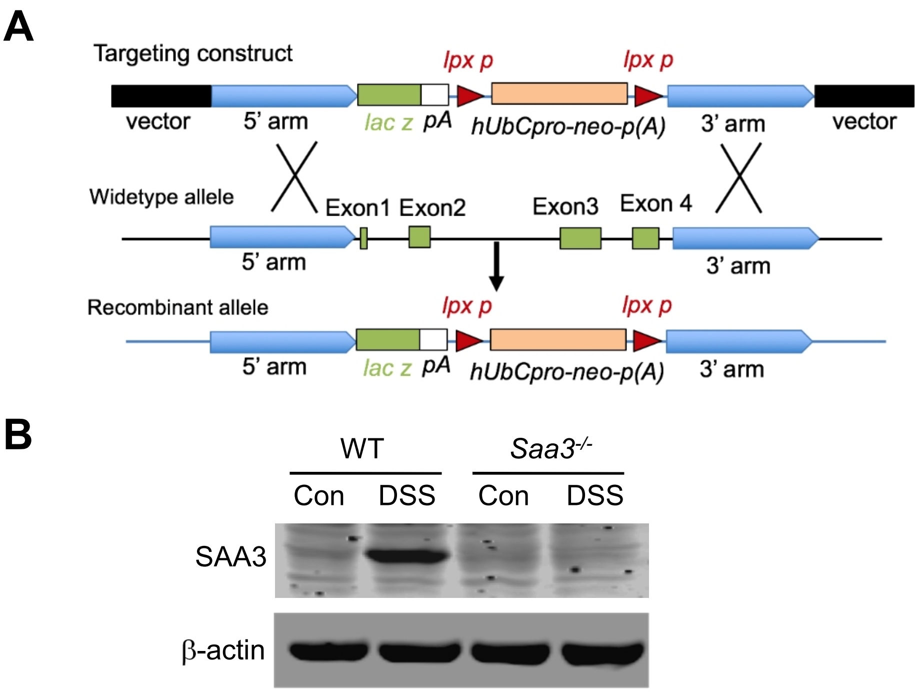

Supplemental Figure S1. Preparation of *Saa3*$^{-/-}$ mice. (**A**) Schematic representation of the Saa3 gene deletion strategy. See *Methods* for detail. (**B**)) Immunoblotting analysis showing colonic mucosal expression of SAA3 after DSS treatment in WT but not *Saa3*$^{-/-}$ mice. Five WT and *Saa3*$^{-/-}$ mice in each group were treated for 7 days with water only (Con) or 3.5% DSS in water (DSS). Colonic tissue was obtained for the preparation of protein samples that were analyzed by SDD-PAGE and Western blotting with an anti-SAA3 antibody. Similar results were obtained, and a representative blot is shown.
