# Supplementary material for: Elevated Expression of Serum Amyloid A 3 Protects Colon Epithelium Against Acute Injury Through TLR2-Dependent Induction of Neutrophil IL-22 Expression in a Mouse Model of Colitis
Source: Front Immunol. 2018 Jun 29;9:1503. doi: 10.3389/fimmu.2018.01503 (PMC6033967; doi:10.3389/fimmu.2018.01503)
Supplement: Supplementary file 2 [file Image_2.PDF]

Supplemental Figures S2

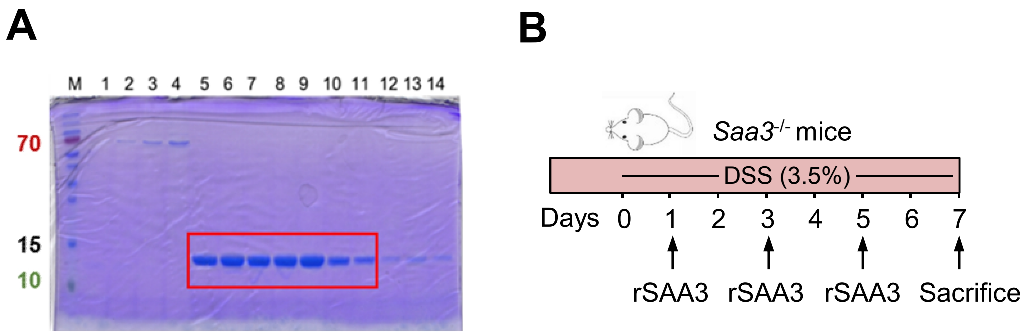

Supplemental Figure S2. Preparation of *Saa3*$^{-/-}$ mice. (**A**) Elusion profile of purified rSAA3 protein with expected size, seen on SDS-PAGE gel with Coomassie brilliant blue staining. Fractions 5-9 were collected for further use. (**B**) A scheme for rSAA3 administration to *Saa3*$^{-/-}$ mice, with rSAA3 (1 µg/kg body weight) given *i.p.* on days 1, 3 and 5 after providing DSS in drinking water. The mice were sacrificed 7 days after DSS treatment for further analysis.
